# Aldehyde dehydrogenase 1 (ALDH1) expression is an independent prognostic factor in triple negative breast cancer (TNBC)

**DOI:** 10.1097/MD.0000000000006561

**Published:** 2017-04-07

**Authors:** Fei Ma, Huihui Li, Yiqun Li, Xiaoyan Ding, Haijuan Wang, Ying Fan, Chen Lin, Haili Qian, Binghe Xu

**Affiliations:** a Department of Medical Oncology, National Cancer Center/Cancer Hospital,Chinese Academy of Medical Sciences and Peking Union Medical College; b Department of Medical Oncology, Shandong Cancer Hospital/Institute, Jinan, Shandong; c Department of Medical Oncology, Beijing Ditan Hospital, Capital Medical University; d State Key Laboratory of Molecular Oncology, Cancer Hospital, Chinese Academy of Medical Sciences and Peking Union Medical College, Beijing, China.

**Keywords:** aldehyde dehydrogenase1, immunohistochemistry, triple negative breast cancer

## Abstract

Triple negative breast cancer (TNBC) is a subset of breast cancer that is highly aggressive and has a poor prognosis. Meanwhile, cancer stem cells (CSCs) are also characterized by a strong tumorigenic potential, which might be partly responsible for the aggressive behavior of TNBC. We previously showed that CSCs are enriched in TNBC cell lines and tissues. Further experiments in animal models revealed higher tumorigenicity of CSCs sorted from TNBC cell lines. In this study, we aimed to determine the clinical relationship between CSCs and TNBC by exploring the expression of aldehyde dehydrogenase 1 (ALDH1), which is a putative marker of breast CSCs, in TNBC tissues.

ALDH1 levels in paraffin-embedded tumor tissues from 158 TNBC patients were evaluated by immunohistochemistry staining using an ALDH1A1 primary antibody. Staining evaluation was performed independently by two pathologists, and the expression level of ALDH1 was evaluated in terms of the percentage and intensity of positive cells. The association of immunohistochemistry staining of ALDH1 expression with clinical parameters was also analyzed.

ALDH1 expression in tumor cells was observed in 88 out of 158 cases (55.7%). Analysis of clinicopathological parameters showed that the immunohistochemistry staining of ALDH1 was significantly correlated with tumor size (*P* = 0.02) and stage (*P* = 0.04). Survival analysis in patients with ALDH1 expression demonstrated shorter relapse-free survival (RFS) and overall survival (OS) times (*P* = 0.01; *P* = 0.001). Moreover, Cox multivariate analysis revealed that ALDH1 expression was an independent prognostic indicator of RFS and OS (*P* = 0.04; *P* = 0.04).

Immunohistochemistry staining of ALDH1 in tumor cells is an independent prognostic indicator of RFS and OS in TNBC patients.

## 1 Introduction

The lack of estrogen receptor (ER), progesterone receptor (PR), and human epidermal growth factor receptor 2 (HER2) expression by triple negative breast cancer (TNBC) tumors confers distinct clinical characteristics on this type of breast cancer, particularly aggressive clinical behavior, early recurrence and metastasis, as well as a worse prognosis.^[1,2]^

Cancer stem cells (CSC) are a tumor cell subset that has a high capacity for self-renewal and tumor initiation, which suggests that CSCs might be partly responsible for the more aggressive behavior of TNBC. In our previous studies, we initially found that CSCs were enriched in TNBC tissues and cell lines relative to other breast cancer subtypes. By sorting and culturing CSCs from TNBC cell lines, we further found that CSCs have higher proliferation and migration rates, and enhanced invasion capacity. Injection of CSCs into the axillary region of NOD/SCID mice demonstrated the stronger tumorigenicity of CSCs from TNBC cell lines.^[3,4]^ These findings led us to hypothesize that the malignant behavior of TNBC might be due to CSCs—a possibility that has not been widely explored. A number of studies showed that aldehyde dehydrogenase 1 (ALDH1) is a specific indicator for identifying, isolating, and tracking human breast CSCs.^[5–10]^ Here, we investigated the relationship between CSCs and TNBC in clinical practice using ALDH1 as a breast CSC marker. We analyzed the clinicopathological parameters of ALDH1 expression in TNBC tumor samples and the prognostic significance of ALDH1 expression.

## 2 Materials and methods

### 2.1 Patients and tumors

Our study was approved by the Institutional Review Board of the Chinese Academy of Medical Sciences Cancer Hospital (No: CH-BC-019). A cohort study was conducted involving 158 patients who were histologically diagnosed with TNBC at the Cancer Hospital of the Chinese Academy of Medical Science between January 2004 and December 2007. All cases were selected based on the availability of clinical data, paraffin blocks, original histological diagnosis of invasive breast carcinoma, and previous pathology reports on TNBC. “Triple negative” was defined as “negative for estrogen receptor (ER; cut-off 10%) and progesterone receptor (PgR; cut-off 10%), as well as HER2 negative (HercepTest: score 0, 1+, and 2+).” In cases that scored 2+, the absence of HER2 gene amplification was confirmed through fluorescence in situ hybridization analysis with the PathVysion kit (Abbott-Vysis, Downers Grove, IL). Records for all 158 patients (median age: 51 years; range: 24–78 years) were examined to gather details of their initial presentation and subsequent treatment. Information on local and disseminated recurrence of disease and the overall survival was also obtained for all patients. The median follow-up time was 55 months (range: 8–160 months). None of the patients exhibited signs of distant metastases at the time of surgery. Adjuvant chemotherapy (anthracycline and/or taxane in most cases) was given to 140 patients (88.6%), whereas neoadjuvant chemotherapy and radiotherapy were given to 21 patients (13.3%) and 65 patients (41.1%), respectively. Breast cancer recurred in 67 patients (42.4%), and 40 patients (25.3%) died from metastasis during the follow-up period.

### 2.2 Immunohistochemistry

Briefly, slides were deparaffinized in xylene and rehydrated in diluted ethanol. Tris-EDTA retrieval buffer (pH 9.0) was used in heat-induced antigen retrieval. After the buffer was cooled to room temperature, endogenous peroxidase activity was blocked with a buffer containing 3% H_2_O_2_ peroxide. Slides were incubated with an ALDH1A1 primary antibody (ab51028, 1:150 dilution; Abcam PLC, Cambridge, MA) for 1 hour at room temperature. After incubation with Envision+ Rabbit Polymer (Dako, CA) for 20 minutes at room temperature, slides were visualized with diaminobenzidine (Dako), lightly counterstained with hematoxylin (Dako), and mounted. Staining specificity was confirmed using liver tissues as the positive control, and Phosphate Buffered Saline instead of the primary antibody was used for a negative control.

### 2.3 Evaluation of staining

Staining evaluation was performed independently by 2 pathologists who recorded ALDH1 expression in tumor cells. ALDH1 protein was mainly located in the cytoplasm with a brownish-yellow diffuse distribution. Results were evaluated by estimating the percentage (*P*) of tumor cells showing characteristic staining (from undetectable level or 0%, to homogeneous staining or 100%) and by estimating the intensity (*I*) of staining.^[11,12]^ The intensity was ranked in ascending order: 0 (no expression), 1 (weak expression), 2 (moderate expression), and 3 (strong expression). Results were scored by the quick score (*Q*) (*Q* = *P* × *I*). Due to the relatively large number of positive cases, a statistical analysis was performed with a negative (score ≤ 10) and positive (score > 10) cutoff. ER or PgR positivity was defined as nuclear staining in more than 10% of carcinoma cells regardless of staining intensity.^[13]^ The cut-off value for HER2 was 10%.^[14,15]^

### 2.4 Statistical analysis

SPSS software (Version 16.0) was used for all statistical analyses. Correlations between tumor cell ALDH1 expression with clinicopathological parameters were assessed through standard chi-squared test or Fisher exact test. Survival analyses were performed for both tumor and stromal cells as appropriate using the Kaplan–Meier method. Relapse-free survival (RFS) and overall survival (OS) were calculated during the period from the date of initial surgery to the date of clinical or pathological relapse or death. Differences in RFS and OS were tested through a log-rank test and Cox proportional-hazards model. All tests were two-sided, and *P* < 0.05 was considered to be statistically significant. The survival rates and hazard ratios were presented as confidence intervals (CIs).

## 3 Results

### 3.1 Expression of ALDH1 protein in tumor cells within tumor tissues

**Figure 1:**
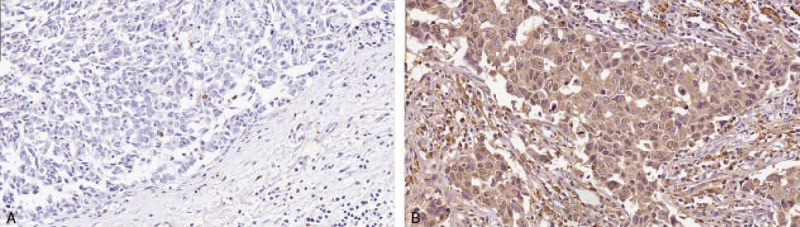
Immunohistochemical staining of aldehyde dehydrogenase 1 (ALDH1) in formalin-fixed, paraffin-embedded breast cancer tissues. A: Local positive staining in tumor cells. B: Diffuse positive staining in tumor cells.

Markedly heterogeneous ALDH1 immunoreactivity was observed in the cytoplasm of tumor cells within tumor tissues (Fig. 1). Among the 158 TNBC cases, ALDH1 expression was observed in 88 cases (55.7%). Most of these cases showed only focal positive expression, while only a few cases showed moderate to strong expression. The proportions of ALDH1-positive cells were: 10–25%, 51 cases; 25–50%, 28 cases; and >50%, 9 cases.

### 3.2 Correlation of ALDH1 expression with clinicopathological parameters in TNBC patients

To assess the potential role of ALDH1 as a prognostic marker for TNBC, we examined whether ALDH1 protein expression correlated with the clinicopathological parameters in TNBC patients. The expression of ALDH1 protein evaluated by immunohistochemistry staining in tumor cells significantly correlated with larger tumor size (*P* = 0.02) and advanced stage (*P* = 0.04) (Table 1). However, there was no significant correlation between ALDH1 expression and other clinicopathological parameters including age, lymph node status, histopathology, grade, tumor embolus, and relapse (Table 1).

**Table 1:**
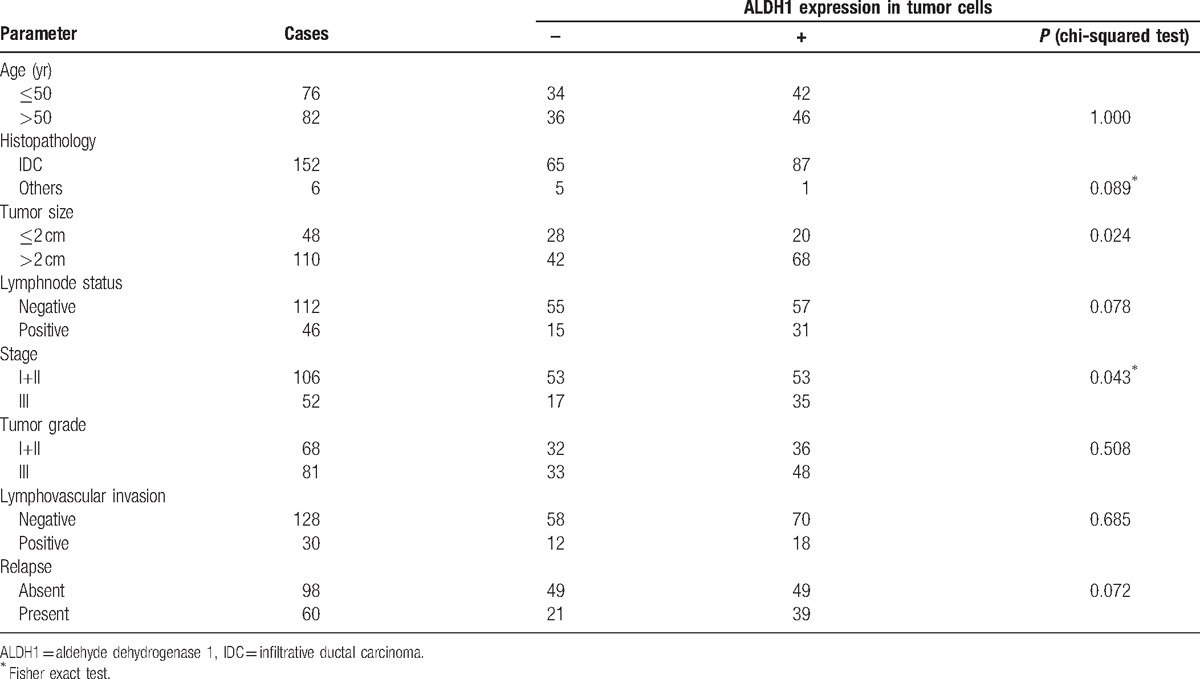
Correlation of ALDH1 expression and clinicopathological parameters in patients with triple negative breast cancer.

### 3.3 ALDH1 protein expression and clinical outcome

**Figure 2:**
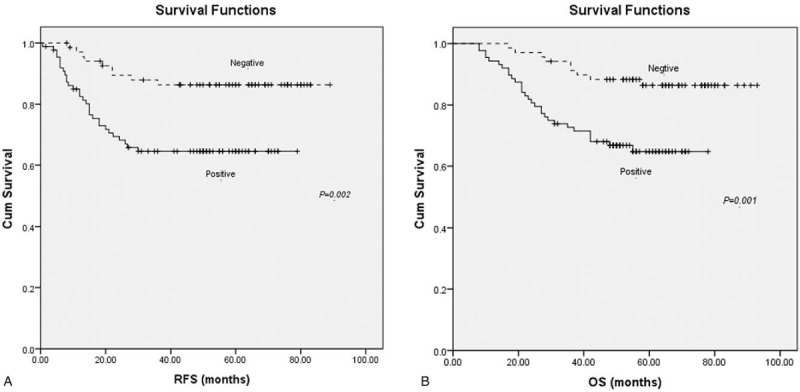
Kaplan–Meier curves for relapse-free survival (RFS) (A) and overall survival (OS) (B) according to aldehyde dehydrogenase 1 (ALDH1) expression status in tumor cells. There were 88 patients in the positive group (lower curve) and 70 patients in the negative group (upper curve).

Survival analysis including log rank testing and Kaplan–Meier analysis demonstrated lower RFS and OS in patients with ALDH1 expression (*P* = 0.01, *P* = 0.001; Fig. 2). The 5-year RFS was 50.7% (95% CI 44.0–57.4) and 70.7% (95% CI 65.1–76.3) for patients with and without ALDH1 protein expression, respectively, while the 5-year OS was 64.6% (95% CI 59.3–69.9) and 86.6% (95%CI 82.4–90.8) for patients with and without ALDH1 protein expression, respectively. According to a Cox multivariate analysis, ALDH1 protein expression was significantly related with RFS (*P* = 0.04) and OS (*P* = 0.04). Together these results indicate that the immunohistochemistry staining of ALDH1 expression in tumor cells was an independent prognostic indicator of RFS and OS (Table 2).

**Table 2:**
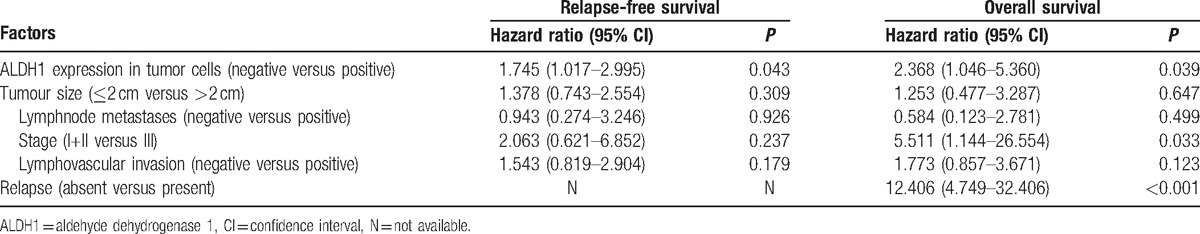
Multivariate analysis of various prognostic factors in triple-negative breast cancer using the Cox proportional hazards model.

## 4 Discussion

Triple negative breast cancer is a breast cancer subtype that is characterized by poor prognosis and aggressive behavior. Meanwhile, cancer stem cells are believed to contribute to tumorigenesis and metastasis. Our earlier studies in cell lines and animal models revealed that CSCs were enriched in TNBC patients. In addition, CSCs sorted from TNBC cell lines were more tumorigenic relative to non-CSCs.^[3,4]^ In the present study, we further analyzed the relationship between CSCs and cancer development in TNBC patients using ALDH1 as a CSC marker. Since the first report by Ginestier et al^[11]^ showing that ALDH1 expression was associated with poor clinical outcome in breast cancer, several studies have indicated that ALDH1+ breast cancer cells are related to TNBC.

In our cohort of 158 TNBC cases, the results of immunohistochemistry staining of ALDH1 revealed that tumor tissues from 55.7% of patients showed ALDH1 expression, which is similar to the rate reported by Ohi et al,^[16]^ but differs from that seen by De Brot et al^[17]^ Interestingly, in our study a majority of the ALDH1+ cases showed low levels of ALDH1 expression that presented as focal positive expression, which may be compatible with the idea that cancer stem cells represent only a minority of the tumor cell population.

As for clinicopathological parameters, we found that the immunohistochemistry staining of ALDH1 expression by tumor cells was significantly correlated with larger tumor size (*P* = 0.024) and later stage (*P* = 0.043), but not with other features including age, lymph node status, histopathology, grade, tumor embolus, and relapse. Although several studies have also addressed the relationship between ALDH1 expression and clinical outcomes, they produced controversial outcomes. In recent meta-analyses of breast cancer studies,Ying et al^[18]^ examined 15 publications and found that ALDH1 expression was associated with larger tumor size, higher histological grade, later stage, higher HER2 expression and lower ER expression, which is in accordance with our results. On the other hand, an analysis by Jin-Fang et al^[19]^ that included 21 eligible studies found a significant association between ALDH1 expression and both histological grade and ER expression but no clear correlation between ALDH1 expression and age, tumor size, lymph node status, lymphovascular invasion, and HER2 expression.

In addition, in this study we found a significant correlation between ALDH1 protein expression in tumor cells and both RFS and OS, suggesting a possible prognostic role for ALDH1 in TNBC. Indeed, Ying et al^[18]^ reported the ALDH1 expression was a biomarker to predict poor survival of breast cancer patients. However, Morimoto et al^[20]^ found that DFS and OS of TNBC patients showed no statistical differences between ALDH1+ and ALDH1− cases. Meanwhile, Resetkova et al^[21]^ showed that stromal but not tumoral expression of ALDH1 was associated with TNBC survival. Although the reasons underlying the discrepancies among these studies are unclear, they may be due to diverse case characteristics, as well as differences in detection sensitivity and evaluation criteria, sample sizes, and follow-up duration. The small number of relevant studies may also explain the lack of consistency in outcomes. Regardless of the reasons for the differences, the absence of consensus suggests that using the expression level of a single molecule to assess disease development in TNBC patients might be inadequate to fully reflect the disease scope.

In conclusion, immunohistochemistry staining of ALDH1 in tumor cells is an independent prognostic indicator of RFS and OS in TNBC. Further studies involving a larger cohort and using standardized and well-matched controls will be needed to verify these results.

## Acknowledgments

None.
